# Advances in Culturomics Research on the Human Gut Microbiome: Optimizing Medium Composition and Culture Techniques for Enhanced Microbial Discovery

**DOI:** 10.4014/jmb.2311.11024

**Published:** 2024-02-19

**Authors:** Hye Seon Song, Yeon Bee Kim, Joon Yong Kim, Seong Woon Roh, Tae Woong Whon

**Affiliations:** 1 Division of Environmental Materials, Honam National Institute of Biological Resource (HNIBR), Mokpo 58762, Republic of Korea; 2 Kimchi Functionality Research Group, World Institute of Kimchi, Gwangju 61755, Republic of Korea; 3 Microbiome Research Institute, LISCure Biosciences Inc., Gyeonggi-do 13486, Republic of Korea

**Keywords:** Culturomics, gut microbiome, cultivation conditions, medium optimization, cultivation techniques

## Abstract

Despite considerable advancements achieved using next-generation sequencing technologies in exploring microbial diversity, several species of the gut microbiome remain unknown. In this transformative era, culturomics has risen to prominence as a pivotal approach in unveiling realms of microbial diversity that were previously deemed inaccessible. Utilizing innovative strategies to optimize growth and culture medium composition, scientists have successfully cultured hard-to-cultivate microbes. This progress has fostered the discovery and understanding of elusive microbial entities, highlighting their essential role in human health and disease paradigms. In this review, we emphasize the importance of culturomics research on the gut microbiome and provide new theories and insights for expanding microbial diversity via the optimization of cultivation conditions.

## Introduction

As a complex ecosystem crucial for various physiological processes, the human gut microbiome has been studied extensively. Over the past decade, advances in next-generation sequencing technologies, particularly metataxonomics (amplification and sequencing of marker genes such as bacterial 16S rRNA genes) and metagenomics (shotgun sequencing of DNA extracted from samples), have revolutionized our understanding of this complexity, revealing the extensive diversity and functionality of microbial entities within the gut [1]. However, despite these technological advancements, certain inherent limitations of microbiology remain, most notably our inability to culture and functionally validate new or rare microbial strains.

Emerging research has revealed the important role of microorganisms in the onset and progression of metabolic diseases, such as obesity, diabetes, and inflammatory bowel diseases, which are influenced by the microbial composition of the gut [2, 3]. Despite the isolation of numerous gut microorganisms, the cultivation of specific, rare gut microbes remains a formidable challenge as highlighted by the fact that up to 70% of the species in the Unified Human Gastrointestinal Genome database have not been cultured to date [4]. In light of these challenges, microbial culturomics emerges as an innovative discipline that focuses on the isolation and cultivation of organisms that are difficult to culture using conventional methods and spearheads advancements in novel cultivation methods [5].

One major aspect of culturomics is its focus on improving cultivation strategies to mimic the complex environmental conditions under which these organisms naturally proliferate. The aim is to enhance cultivation methods by integrating diverse culture media, adjusting temperature and atmospheric parameters, and simulating the intricate microbial interactions observed in the organisms’ native habitat. Cultivation efforts accompanied by genomic analysis allow for the rapid identification and characterization of isolated microorganisms. The process provides valuable insights into the functional roles, metabolic pathways, and potential applications of microbes in various fields such as biotechnology, medicine, and environmental science. Microbial cultivation, such as the cultivation of anaerobic or fastidious microorganisms, present continuous challenges for researchers but the emergence of novel molecular biology techniques heralds unprecedented possibilities in this field [6]. Metagenomic analyses have revealed that the gut hosts a more diverse microbial community than was previously believed [7]. Culturomics alongside metagenomic analyses enables us to understand microbes’ influence on human health, thus paving the way for groundbreaking developments in personalized medicine and microbial therapies [8]. In this review, we discuss recent advances and limitations of culturomics in the study of the gut microbiome.

## Challenges in 16S rRNA Gene and Metagenome Analyses in Microbiome Research

A major limitation of 16S rRNA gene sequence analysis is that it is difficult to distinguish closely related microbial species, which limits our understanding of the diversity and functions of the microbial community [9]. The relationship between *Escherichia coli* and *Escherichia fergusonii* illustrates this issue. *E. coli*, commonly found in the human gut, plays an essential role in digestion and immune functions, whereas *E. fergusonii*, although genetically similar to *E. coli* with a DNA-DNA hybridization similarity of approximately 64% [10], has been less studied and is not well understood [11, 12]. Since certain strains of *E. coli* can be harmful while others can be beneficial, accurate identification and differentiation within the genus *Escherichia* is critical for the reliability of microbiome research. This problem is not limited to the *Escherichia* genus. Microbes belonging to the genus *Shigella* share a close phylogenetic relationship with the *Escherichia* genus and have similar 16S rRNA gene sequences (>99% sequence identity) [13], making accurate differentiation challenging. *Shigella* is a known pathogen, and the inability to differentiate between these two genera could lead to significant misunderstandings in microbiome research.

Furthermore, most bacterial species possess multiple rRNA operons (1–27 16S-23S-5S rRNA operon copy numbers) [14], which can distort 16S rRNA gene analysis. This affects data interpretation and the quantitative assessment of microbial communities. Although metagenomic analysis offers several broad insights, its implementation is challenging. Metagenomic data is highly complex and requires advanced computational capabilities and sophisticated tools for its analysis, alongside high sequencing costs [15]. Thus, it is less accessible to smaller laboratories or institutions with limited resources. Additionally, large DNA samples are necessary for metagenomic analyses, which may not be practical in studies with limited sample sizes.

## The Unveiling of Unculturable Bacteria: A Leap Forward in Microbial Ecology

Microbial dark matter comprises bacteria that scientists are not yet able to culture and remains a significant frontier in microbiological research. These elusive members of the microbial community, often referred to as the ‘most wanted taxa,’ are pivotal in deciphering microbial diversity and functions that have been shrouded in mystery because of our limited ability to cultivate them using conventional methods [16]. Groundbreaking strides have been made with innovative culturing techniques that have led to the growth of previously unculturable bacteria in the lab, thus bridging critical gaps in our understanding of microbial ecology. The anaerobic culture system, especially the development of an anaerobic chamber, allows researchers to grow important obligate anaerobes of the human gut, such as *Faecalibacterium* and *Akkermansia* [17, 18]. Another noteworthy example of culturing previously unculturable bacteria is the discovery of *Muribaculum intestinale*. This species is a cryptic member of the *Muribaculaceae* family, which is primarily found in the intestines of mammals and was previously referred to as ‘S24-7.’ This population has been challenging to culture and has eluded microbiologists for a long time. However, Lagkouvardos *et al*. [19] successfully isolated and cultured some members of the *Muribaculaceae* family using enhanced culturing methods. These discoveries have enabled a new understanding of the physiology and function of previously difficult-to-culture microbial populations, potentially providing deeper insights into their roles in human health and disease [20, 21].

**Fig. 1:**
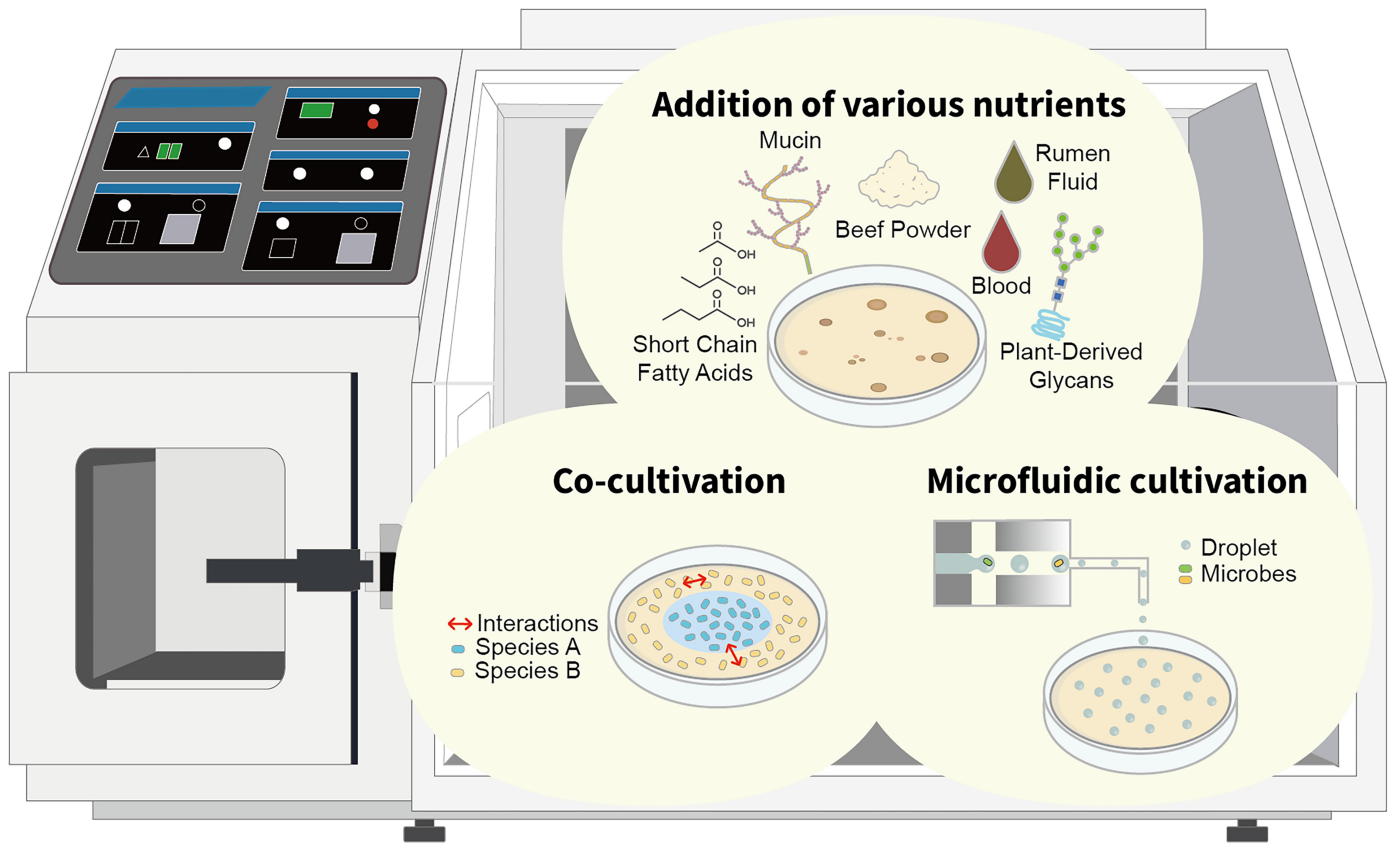
Advanced cultivation methods for isolating human gut microbes.

These successes emphasize the importance of culturomics research in understanding complex microbial ecosystems, underscoring this as an essential step in developing culturing conditions that accurately reflect microbial diversity and complexity. Overlooked microbes may act as keystone species that have a disproportionate impact on community structure and function despite their low abundance. Their presence and function could be crucial to the complexity and dynamics of human gut microbiota. To circumvent these limitations, it is imperative to advance and refine current microbiome research techniques (Fig. 1).

## Strategic Cultivation Medium Composition for Gut Microbial Research

### Types of Media for Cultivating Intestinal Microorganisms

Culturomics can overcome the limitations of traditional cultivation methods, facilitating comprehensive research on challenging-to-culture gut microbes and enhancing our understanding of microbial ecosystems in the gut. Gut microbes require specific nutritional and environmental conditions, making a strategic cultivation media composition essential for supporting the growth and development of these microbes. Commonly used non-selective media (Columbia blood agar, brain heart infusion, Columbia nalidixic acid, phenylethyl alcohol, gut microbiota medium, fastidious anaerobe broth, Choco, Choco-pasteurized, and Gifu Anaerobic Medium) support the selective growth of specific microbial species, whereas others require different optimized conditions [22, 23]. Examples of media compositions used for the isolation of human gut microbes are shown in Table 1.

Previous research has suggested that many gut bacteria that were previously considered uncultivable can be cultured in commercially available media [22]. However, Fleming *et al*. [24] reported that approximately 18.1% of the genera from fecal sample could be isolated, representing approximately 67.9% of the communities predicted by metagenomic analyses. With the development of advanced culture techniques and methods, the proportion of cultured microorganisms is likely to increase, further enriching our understanding of the gut microbiome.

### Diversity of Intestinal Microorganisms under Different Nutritional Conditions

Each microorganism has different nutritional and environmental requirements, and thus the culture media should be carefully designed to enhance the success of microbial isolation and growth. Various attempts have been made to separate gut microorganisms effectively, each utilizing different media compositions, resulting in different success rates [22, 25-27]. Many studies reflect the diverse growth potential of microbial populations in the gut environment.

Using culturomics approaches, a range of new bacterial species and strains have been discovered. One study successfully isolated 106 bacterial species from five fecal samples, of which three were novel species, and six had not been previously isolated from the human body [28]. To maximize the effectiveness of culturomics, it is crucial to determine optimal culture conditions, and various culture conditions and techniques are currently under evaluation. A recent study tested over 300 different culture conditions and used 58 of them to isolate 497 bacterial species from eight fecal samples [26], concluding that the number of conditions used could be reduced by more than half.

Furthermore, a recent study found that the success of microbial community culture from infants’ fecal samples was markedly influenced by the specific growth medium formulation used [27]. Each bacterial species in the samples demonstrated different growth requirements and performance depending on the culture medium. This study also analyzed the correlation between bacterial growth and various media components, revealing that several bacterial taxa were positively correlated with complex plant-derived glycans and simple carbon sources, whereas other taxa required chemically undefined substances, such as tryptone, casein, peptone, and yeast extract, for growth and proliferation. This diversity and specificity of nutritional requirements emphasizes the importance of culture medium composition.

### Optimizing Cultivation Conditions for Intestinal Microorganisms

Meeting the nutritional and environmental needs of microorganisms requires thoughtful selection of medium components such as carbohydrates, nitrogen compounds, vitamins, and minerals [29, 30]. For example, carbohydrates act as energy sources, while nitrogen compounds are fundamental for protein metabolism. Vitamins and minerals are vital for the activation of enzymes and for sustaining cellular functions. Through the strategic configuration of culture media and by providing the breadth of necessary nutritional elements, researchers can optimize the growth and isolation of intestinal microorganisms. In this endeavor, it is imperative to delve deep into studying and analyzing various media conditions and their impact, fostering a nuanced understanding of their effects on microbial cultivation. Thus, a refined understanding facilitated by carefully orchestrated culturomics strategies is essential.

### Exploring Additional Nutritional Strategies and Microbial Interactions

Research is continuously underway to customize and standardize protocols for the cultivation of various gut microbes [24, 26, 31]. A blood-enriched medium typically containing 5% blood mimics the nutritional conditions of the host and promotes the growth of microbes adapted to the host environment [32, 33]. Serum acts as a growth factor by providing numerous elements such as lipids, vitamins, neutral fats, and minerals [32]. Rumen fluid, which is rich in diverse microbes and digestive enzymes, provides a conducive environment for anaerobic bacteria. The addition of rumen fluid resulted in a significant increase in the number of isolated bacterial species compared to the number of bacteria isolated under conditions without rumen, proving that the rumen is necessary for cultivating a substantial number of bacterial species [26]. Additionally, beef powder, which is rich in essential proteins and amino acids, plays a crucial role as a nutritional source for promoting microbial growth. The presence or absence of beef powder is suggested to be a decisive factor in determining the rate and success of microbial growth [34, 35]. Mucin, the main component of the mucus layer in the gastrointestinal tract, provides an environment that mimics the host gut. Its incorporation into the culture media supports the growth of gut-associated microbes such as *Akkermansia muciniphila*, which remarkably utilizes mucin as the sole source of carbon and nitrogen [36]. This unique metabolic adaptation allows *A. muciniphila* to thrive in mucin-rich environments, supporting its role in maintaining gut barrier integrity and overall gut health. Likewise, *Bacteroides thetaiotaomicron*, a species ubiquitously present in the human gut, flourishes in the presence of mucin [37, 38]. This highlights the importance of optimizing the culture media by incorporating mucin to accurately mimic the natural gut environment. Such optimization facilitates the study of the physiological roles and interactions of this microorganism within the complex gut ecosystem.

Gut microbes exist interdependently, requiring various nutrients mediated by bacterial metabolic products such as vitamins, amino acids, and short-chain fatty acids (SCFAs) [39]. Amino acids support microbial protein synthesis, and externally supplied amino acids promote the growth of certain microbes unable to synthesize them directly [40]. Vitamins are crucial elements for microbial cell structure, function, and various metabolic processes. Because certain microbes cannot synthesize vitamins on their own, adding vitamins to the culture medium can support microbial growth and survival [41]. SCFAs serve as an energy source for gut microbial communities and play a vital role in maintaining the health and function of the intestinal mucosa. As microbial metabolic products, SCFAs can provide the specific conditions necessary for the growth and proliferation of microbes that are difficult to cultivate [42]. The hard-to-cultivate microbe *Faecalibacterium* prausnitzii is known to require vitamins (biotin, riboflavin, folic acid, and cobalamin) and volatile fatty acids for growth [43]. Overall, the appropriate combination and provision of these nutrients can improve the cultivation conditions for difficult-to-cultivate gut microbes [44].

## Innovative Cultivation Techniques in Culturomics

### Co-culture Techniques

The landscape of culturomics is continually shaped by innovative technologies aimed at optimizing microbial cultivation. One such advancement is the utilization of co-culture techniques, providing a nuanced exploration of the intricate interactions among diverse microbial species in their natural environment. Unlike traditional culture methods that focus on the isolation and study of a single microbial species, co-culture techniques allow researchers to grow multiple species together, mirroring their natural environments more closely. This approach is particularly beneficial for fastidious gut microbes with complex nutritional requirements, replicating their reliance on metabolites produced by neighboring microbes for optimal growth.

Recent studies have interesting findings that revealed unique symbiotic relationships within microbial communities, particularly within the genus *Faecalibacterium*. *Faecalibacterium* and *Clostridium* IV are the major butyrate-producing microbes in the gut, and their growth is enhanced when they are co-cultured with *Bifidobacterium*, *Phocaeicola*, and *Bacteroides* [45]. These findings suggest that there may be beneficial metabolic interactions in which the latter microbes facilitate the growth of the former by producing metabolites necessary for growth. However, not all of these interactions are mutually beneficial. For instance, *Phocaeicola* can be inhibited when co-cultured with *Faecalibacterium*, representing a unilaterally beneficial interaction. In addition, some microbes promote the growth of *Faecalibacterium*, *Bacteroides*, *Bilophila*, *Gordonibacter*, and *Sutterella* via quinone production [46], indicating that quinone-producing microbes can aid the growth of gut microbes that are difficult to culture. Gamma-aminobutyric acid (GABA) also plays an important role in enhancing the growth of gut microbes [47]. *Evtepia gabavorous* KLE1738, once a highly coveted microbe to culture, was successfully isolated through co-cultivation with *Bacteroides fragilis*, demonstrating a dependency on the presence of GABA-producing microbes. GABA, a carbon and nitrogen source, plays a significant role in promoting the growth and metabolic activity of specific gut microbial species. All these studies contribute significantly to our understanding of the complex interactions and growth mechanisms of gut microbial communities and provide new methods and approaches for microbial cultivation.

### Microfluidics in Microbial Cultivation

Droplet-based microfluidics is a transformative technology that facilitates the handling of liquid volumes at the picoliter to nanoliter scale. This approach has unveiled powerful high-throughput applications across various disciplines, paving the way for remarkable advancements in microbial cultivation [48, 49]. This enables the individual culture of thousands of single-cell droplets, minimizes microbial competition, and allows for the cultivation of low-density or slow-growing microorganisms in the intestinal environment. Consequently, this technology enriches the diversity of microorganisms by fostering the growth of strains that were previously deemed uncultivable using traditional methods.

Furthermore, the advent of single-cell dispensing (SCD) technology has been instrumental in fostering developments in microbial cultivation [50]. Characterized by its high-throughput capabilities and label-free classification mechanisms, SCD has emerged as a potent alternative to classical agar plate technology, enhancing the efficiency of microbial separation and cultivation processes. SCD facilitates a more streamlined and rapid cultivation process, significantly reducing the time required to obtain pure cultures and thus promoting a more comprehensive and efficient exploration of microbial diversity and abundance. Utilizing SCD technology in gut microbiota studies has led to the identification of 82 bacterial species across five phyla and 24 families existing in the human gut, and proposals of 11 new genera and 10 new species [50]. In addition, a novel technology known as growth within double emulsions (GrowMiDE) has been introduced. This methodology allows the cultivation of a diverse array of microbes, including taxa that are unrepresented in traditional batch cultures. For instance, with the prevention of nutrient monopolization by fast-growing microbes in double emulsion droplets, it is possible to cultivate slow-growing *Negativicutes* and *Methanobacteria* in enriched media cultures [51]. These innovative technological strides continue to redefine the boundaries of microbial cultivation, fostering enhanced diversity and a deeper understanding of microbial ecology.

## Conclusion

In conclusion, continuous evolution and improvement of culture methods are paramount for leveraging our understanding of the vast array of microbes inhabiting the human gut. The strategic optimization of culture media components to create customized environments significantly augments the potential for cultivating a diverse spectrum of microbes, including those traditionally deemed challenging to culture. Such meticulous approaches not only reveal previously undiscovered microbial species and lineages, but also enhance our understanding of microbial diversity and its intricate roles in human health and disease.

## Figures and Tables

**Table 1:** Commonly used media for isolating gut microorganisms from human feces.

Medium	Carbohydrate	Extract and Enzymatic Hydrolysate	Vitamin	Blood	Rumen Fluid	SCFA	Antibiotic	Isolated Microbes (Genus)
5 µm-filtered BB	Glucose Sucrose	Yeast Extract Casein Enzymic Hydrolysate Papaic Digest of Soyabean Meal Animal Tissue Digest	B_6_, K_3_	-	-	-	-	*Acidaminococcus, Actinomyces, Bacillus, Bacteroides, Bifidobacterium, Blautia, Citrobacter, Clostridium, Coprobacillus, Corynebacterium, Dietzia, Eggerthella, Enterococcus, Escherichia, Eubacterium, Facklamia, Flavonifractor, Fusobacterium, Klebsiella, Lysinibacillus, Parabacteroides, Pseudomonas, Ruminococcus, Streptococcus*
BB with Blood	Glucose Sucrose	Yeast Extract Casein Enzymic Hydrolysate Papaic Digest of Soyabean Meal Animal Tissue Digest	B_6_, K_3_	Sheep Blood	-	-	-	*Acidaminococcus, Alistipes, Anaerococcus, Bacteroides, Bifidobacterium, Blautia, Clostridium, Collinsella, Dietzia, Eggerthella, Enterococcus, Escherichia, Flavonifractor, Murdochiella, Odoribacter, Parabacteroides, Peptoniphilus, Pseudomonas, Staphylococcus*
BB with Rumen Fluid	Glucose Sucrose	Yeast Extract Casein Enzymic Hydrolysate Papaic Digest of Soyabean Meal Animal Tissue Digest	B_6_, K_3_	-	+	-	-	*Acidaminococcus, Alistipes, Bacteroides, Blautia, Campylobacter, Candida, Citrobacter, Clostridium, Dietzia, Eggerthella, Enterococcus, Escherichia, Facklamia, Flavonifractor, Klebsiella, Odoribacter, Parabacteroides, Pediococcus, Pseudomonas*
TSB	Glucose	Pancreatic Digest of Casein Peptic Digest of Soybean	-	-	-	-	-	*Bacillus, Corynebacterium, Dietzia, Enterococcus, Escherichia, Facklamia, Klebsiella, Lysinibacillus, Micrococcus, Pseudomonas, Staphylococcus, Streptococcus*
TSB with Blood	Glucose	Pancreatic Digest of Casein Peptic Digest of Soybean	-	Sheep Blood	-	-	-	*Acidaminococcus, Anaerococcus, Bacillus, Bacteroides, Blautia, Citrobacter, Clostridium, Collinsella, Dietzia, Eggerthella, Enterococcus, Escherichia, Eubacterium, Finegoldia, Flavonifractor, Klebsiella, Lactococcus, Lysinibacillus, Odoribacter, Parabacteroides, Peptoniphilus, Peptostreptococcus, Streptococcus*
BHI	Glucose	Brain Heart Infusion Peptic Digest of Animal Tissue	-	-	-	-	-	*Acidaminococcus, Ruminococcus*
BHI with Colistin and Vancomycin	Glucose	Brain Heart Infusion Peptic Digest of Animal Tissue	-	-	-	-	Colistin Vancomycin	*Bacteroides, Candida, Citrobacter, Clostridium, Dietzia, Enterococcus, Flavonifractor, Lactobacillus, Pseudomonas, Pediococcus*
Gut Microbiota Medium	Glucose Fructose Maltose	Yeast Extract Meat Extract Tryptone Peptone	K_3_, ATCC Vitamin Mix	-	-	Acetic Acid Isovaleric Acid Propionic Acid Butyric Acid	-	*Bifidobacterium, Catenibacterium, Enterococcus, Lactobacillus*
Modified Gifu Anaerobic Medium	Glucose Starch	Yeast Extract Meat Extract Liver Extract Peptone Soya Peptone Digested Serum	K_1_	-	-	-	-	*Acidaminococcus*
Mueller Hinton Medium	Starch	Beef Extract Powder Acid Hydrolysate of Casein	-	-	-	-	-	*Acidaminococcus, Ruminococcus*
Phenylethyl Alcohol Medium with Blood	-	Papaic Digest of Soybean Meal Pancreatic Digest of Casein	-	Sheep Blood	-	-	β-Phenylethyl Alcohol	*Acidaminococcus, Ruminococcus*
BY Chocolate Medium	Starch	Yeast Extract Meat Extract Pancreatic Digest of Casein Acid Hydrolysate of Casein Peptic Digest of Animal Tissue	B1	Laky Horse Blood	-	-	-	*Acidaminococcus, Clostridium, Ruminococcus*
Potato Dextrose Medium	Glucose	Potato Infusion	-	-	-	-	-	*Enterococcus, Megasphaera, Mitsuokella*
Lactobacilli MRS Medium	Glucose	Yeast Extract Beef Extract Proteose Peptone (No.3)	-	-	-	Acetic Acid	-	*Anaerostipes, Megasphaera, Mitsuokella*
Mitis Salivarius Medium	Glucose Sucrose	Proteose Peptone (No.3+normal) Pancreatic Digest of Casein	-	-	-	-	Potassium tellurite	*Bifidobacterium, Streptococcus*
CIN Medium	Mannitol	Yeast Extract Beef Extract Pancreatic Digest of Gelatin Peptic digest of Animal Tissue	-	-	-	-	Sodium Deoxycholate Cefsulodin Novobiocin Triclosan	*Fusobacterium*
Listeria Enrichment Medium	Glucose	Yeast Extract Soy Peptone Pancreatic Digest of Casein	-	-	-	-	Acriflavine Cycloheximide Nalidixic acid	*Eubacterium*
CCFA Medium	Fructose	Peptic Digest of Animal Tissue	-	-	-	-	Cefoxitin Cycloserine	*Coprobacillus*
Tomato Juice Medium	-	Peptone Peptonized milk	-	-	-	-	-	*Megasphaera, Mitsuokella*
Drigalski Lactose Medium	Lactose	Pancreatic Digest of Casein Meat Peptic Digest Peptic Digest of Animal Tissue	-	-	-	-	-	*Megasphaera, Mitsuokella*
Selenite Medium	Lactose	Pancreatic Digest of Casein	-	-	-	-	-	*Fusobacterium*
Modified FM Medium	Glucose Starch	Yeast Extract Meat Extract Peptone Soy Peptone Liver Digest Serum Pancreatic Digest	-	-	-	-	Neomycin	*Fusobacterium*
DHL Medium	Sucrose Lactose	Meat Extract Peptone	-	-	-	-	Sodium Deoxycholate	*Fusobacterium*
Modified Peptone- Yeast Extract- Glucose Medium	Glucose	Yeast Extract Beef Extract Trypticase Peptone Peptone	K_1_	-	-	Acetic acid	-	*Faecalibacterium*
Modified Bicarbonate- Buffered Medium	Glucose N-Acetylglucosamine	Soy Peptone	Vitamin Solution	-	+	-	-	*Akkermansia*

*SCFA, short-chain fatty acid; TSB, tryptic soy broth; BB, BD BACTEC Media; BHI, brain heart infusion

Data were collected from the references [17, 18, 21, 24].
